# Acute adaptation of central and peripheral motor unit features to exercise‐induced fatigue differs with concentric and eccentric loading

**DOI:** 10.1113/EP091058

**Published:** 2023-04-05

**Authors:** Eleanor J. Jones, Yuxiao Guo, Eduardo Martinez‐Valdes, Francesco Negro, Daniel W. Stashuk, Philip J. Atherton, Bethan E. Phillips, Mathew Piasecki

**Affiliations:** ^1^ Centre of Metabolism, Ageing and Physiology (COMAP), MRC‐Versus Arthritis Centre for Musculoskeletal Ageing Research, National Institute for Health Research (NIHR) Nottingham Biomedical Research Centre University of Nottingham Nottingham UK; ^2^ Centre of Precision Rehabilitation for Spinal Pain (CPR Spine), School of Sport, Exercise and Rehabilitation Sciences, College of Life and Environmental Sciences University of Birmingham Birmingham UK; ^3^ Department of Clinical and Experimental Sciences Università degli Studi di Brescia Brescia Italy; ^4^ Department of Systems Design Engineering University of Waterloo Waterloo Ontario Canada

**Keywords:** fatigue, high density electromyography, intramuscular electromyography, motor unit

## Abstract

Force output of muscle is partly mediated by the adjustment of motor unit (MU) firing rate (FR). Disparities in MU features in response to fatigue may be influenced by contraction type, as concentric (CON) and eccentric (ECC) contractions demand variable amounts of neural input, which alters the response to fatigue. This study aimed to determine the effects of fatigue following CON and ECC loading on MU features of the vastus lateralis (VL). High‐density surface (HD‐sEMG) and intramuscular (iEMG) electromyography were used to record MU potentials (MUPs) from bilateral VLs of 12 young volunteers (six females) during sustained isometric contractions at 25% and 40% of the maximum voluntary contraction (MVC), before and after completing CON and ECC weighted stepping exercise. Multi‐level mixed effects linear regression models were performed with significance assumed as *P* < 0.05. MVC decreased in both CON and ECC legs post‐exercise (*P* < 0.0001), as did force steadiness at both 25% and 40% MVC (*P* < 0.004). MU FR increased in ECC at both contraction levels (*P* < 0.001) but did not change in CON. FR variability increased in both legs at 25% and 40% MVC following fatigue (*P* < 0.01). From iEMG measures at 25% MVC, MUP shape did not change (*P* > 0.1) but neuromuscular junction transmission instability increased in both legs (*P* < 0.04), and markers of fibre membrane excitability increased following CON only (*P* = 0.018). These data demonstrate that central and peripheral MU features are altered following exercise‐induced fatigue and differ according to exercise modality. This is important when considering interventional strategies targeting MU function.

## Abstract (highlights)

- **What is the central question of this study?**
  Conflicting evidence exists on motor unit (MU) firing rate in response to exercise‐induced fatigue, possibly due to the contraction modality used: Do MU properties adapt similarly following concentric and eccentric loading?
- **What is the main finding and its importance?**
  MU firing rate increased following eccentric loading only despite a decline in absolute force. Force steadiness deteriorated following both loading methods. Central and peripheral MU features are altered in a contraction type‐dependant manner, which is an important consideration for training interventions.

## 1 INTRODUCTION

The motor unit (MU) is a key component of the motor system and control of muscle force is regulated by the rate coding and recruitment of MUs (Enoka & Duchateau, 2017). The concept of fatigue is vast and encompasses several definitions; however, that related to neuromuscular decrement is most accurately described as performance fatigue: the loss of force and/or power output from a muscle as a result of impaired contractile function and/or muscle activation (Enoka & Duchateau, 2016). Performance fatigue is a feature often seen acutely post‐exercise and is commonly the cause of task failure (Hunter et al., 2004). It is also observed in chronic clinical conditions (Burtin et al., 2012; Chaudhuri & Behan, 2004; Prinsen et al., 2015) and ageing (Christie et al., 2011; Merletti et al., 2002). Irrespective of the setting, the involvement of multiple physiological mechanisms and experimental triggers presents challenges in determining the cause (Enoka & Duchateau, 2008).

The muscle response to fatigue has been shown to be affected by the muscle contraction modality. For example, fatigue caused by both concentric (CON) and eccentric (ECC) contractions elicits reductions in muscle strength (Linnamo et al., 2000) and muscle activation (Peñailillo et al., 2013) yet the recovery profiles relating to damage are different (Souron et al., 2018). Of the three commonly applied contraction types (isometric, CON and ECC), ECC ‘lengthening’ contractions generate greater voluntary forces and appear less strenuous due to a lower metabolic cost and cardiovascular stress (Hody et al., 2019; Overend et al., 2000; Webber & Kriellaars, 1997). These features make eccentric training programmes favourable for improving muscle mass and strength in both healthy adults and those with compromised musculoskeletal health (Cook et al., 2013; Roig et al., 2009).

The neural mechanisms influencing performance fatigue, including MU recruitment thresholds, firing rate (FR) and voluntary activation, appear to differ across exercise modalities (Duchateau & Baudry, 2014; Kay et al., 2000) with some data showing an increase in MU FR following fatiguing exercise (Dartnall et al., 2009; Piitulainen et al., 2012), while others report a decrease (Adam & De Luca, 2005; Contessa et al., 2016; Kuchinad et al., 2004; Rubinstein & Kamen, 2005; Stock et al., 2012). More recently, a direct comparison of contraction modalities showed a greater increase in MU FR following CON, when compared to ECC contractions (Hirono et al., 2022). These equivocal findings may be explained by a muscle specific effect or by differences in the way fatigue was induced, with reductions in maximal contraction following prolonged low‐intensity activity occurring as a result of reduced muscle activation, and reductions following high‐intensity activity attributable to impaired contractile function (Enoka & Duchateau, 2016).

High density surface EMG (HD‐sEMG) records multiple MU potentials (MUPs) from a relatively large volume of muscle (Martinez‐Valdes et al., 2016) and is well placed to assess FR of multiple MUs during a single contraction (Negro et al., 2016; Oliveira & Negro, 2021) and at larger contraction intensities (Del Vecchio et al., 2018). Additionally, the use of intramuscular EMG (iEMG) with concentric needles enables a more detailed view of MUPs via sampling without the limitations of signal attenuation through skin and subcutaneous tissue. iEMG recordings enable estimation of neuromuscular junction (NMJ) transmission instability and temporal dispersion across MU propagating action potentials (Piasecki et al., 2021) as well as MUP gradients reflecting ion exchange and membrane excitability. Thus, the combination of these techniques allows an overall assessment of central and peripheral MU adaptations to a given stimulus. The aim of this study was to determine the muscle‐specific response of vastus lateralis (VL) MU features following a functional stepping task employing bilateral CON and ECC movements performed to failure. It was hypothesised that strength and force steadiness would decline in both legs as a result of performance fatigue. Due to the higher metabolic demand of CON movements, it was hypothesised MU function would be more greatly affected by CON movements than ECC.

## 2 METHODS

### 2.1 Ethical approval

Seventeen healthy recreationally active volunteers (nine female, eight male) gave written informed consent to take part in this study, which was approved by the University of Nottingham Faculty of Medicine & Health Sciences Research Ethics Committee (186‐1812). The study was conducted in accordance with the *Declaration of Helsinki* except for registration in a database. Data are available for 12 participants (six female, six male) for iEMG, and 10 participants (five female, five male) for HD‐sEMG due to low yield of MUs at one or more time points and/or contraction levels. Exclusion criteria included a body mass index (BMI) <18 or >35 kg/m^2^, currently competing in sports at regional level or above, or a history of cardiovascular, respiratory or neuromuscular disorders. All participants were asked to refrain from strenuous exercise 48 h prior to assessment. All participants were medically screened prior to the study via a medical history questionnaire and a resting electrocardiogram. Body mass and height were measured using calibrated scales and stadiometry, respectively, and BMI was calculated.

### 2.2 Experimental protocol

#### 2.2.1 Fatigue protocol

To induce simultaneous CON and ECC fatigue in all participants, we employed a step up, step down protocol (Kostek et al., 2007). Participants concentrically contracted the quadriceps by stepping up onto a 43.5 cm‐high bench with one leg, and stepping down with the opposing leg eccentrically, with each stepping contraction timed to a 3 s metronome. Participants wore a weighted vest (initially 25% of body weight but increasing up to 40% depending on tolerance) during the intervention. The task was performed until self‐ascribed exhaustion, which was indicated as 10 using a modified Borg scale. The average time to exhaustion was 53 ± 12 min, with 60 s timed rest stop permitted when requested, for a maximum of three rest stops. All participants reported they were right‐leg dominant, and the leg assigned to CON or ECC was randomised, as was the order in which the post‐stepping assessments were conducted. All assessment procedures were performed immediately before the fatiguing protocol, and post‐testing began on the first limb within 3 min following completion of the protocol. All electrodes remained in place secured by tape between pre‐ and post‐testing, so reapplication was not required. Assessment of the second limb began approximately 12 min later.

#### 2.2.2 Strength assessment

Knee extensor strength was assessed with participants sitting with hips and knees flexed at 90° and the leg securely fastened to a force transducer just above the ankle. To familiarise with the equipment and ‘warm‐up’ the muscle, participants performed a series of submaximal contractions. They were then instructed to perform a maximal isometric contraction, accompanied by verbal encouragement and visual feedback of force. This was repeated three times, with 60 s rest intervals. The best effort was taken as maximum voluntary isometric contraction force (MVC). After determining MVC, participants performed isometric voluntary contractions each lasting 12−15 s, aiming to hold a target line set at 25% and 40% MVC displayed on a monitor. Following a familiarisation trial, HD‐sEMG and iEMG were recorded simultaneously, with rest for ∼30 s between contractions. Voluntary contraction and signal recording was repeated until a minimum of six recordings at 25% and three at 40% had been obtained. Contractions were performed alternating between 25% and 40% MVC. Post‐intervention forces were normalised to the post‐intervention MVC. The coefficient of variation of the force trace (CoV force) represented force steadiness. The force signals were displayed in real‐time using Spike2 software (v9.06, Cambridge Electronic Design, Cambridge, UK) and data collected simultaneously from the force transducer for offline analysis. EMG data was collected during each of these contractions as detailed below.

#### 2.2.3 HD‐sEMG

A semi‐disposable adhesive monopolar HD‐sEMG matrix (GR08MM1305, OT Bioelettronica, Torino, Italy) consisting of 64 × 8 mm spaced electrodes was positioned in the presumed direction of the fascicles on both (right and left) VLs and secured to the skin before the pre‐fatigue assessments. This remained in situ until after the post‐assessments. A reference cable (CPAT1, OT Bioelettronica) was attached around the ankle of the recording limb. The signal was amplified, sampled at 2000 Hz, band‐pass filtered (10–500 Hz) and converted to digital data (16‐bit analog–digital converter, 3 dB bandwidth) using a Sessantaquatro (OT Bioelettronica) multi‐channel amplifier. Data were visualised in real‐time in OTBioLab+ software (v1.3.2, OT Bioelettronica) and stored for offline analysis.

#### 2.2.4 Intramuscular EMG

A 25 mm concentric needle electrode (74025‐45/25 Neuroline; Ambu, Baltorpbakken, Denmark) was inserted directly above the HD‐sEMG electrode. A voluntary, low force contraction was performed by the participant while the needle position was adjusted to ensure its tip was close to fibres belonging to active MUs (Piasecki et al., 2019; Stashuk, 1999a). The participant then performed isometric voluntary contractions lasting 12−15 s, aiming to hold a target line set at 25% and 40% MVC as described above. The needle electrode was repositioned between contractions by combinations of rotating the bevel 180° and withdrawing it by 10–25 mm to sample MUs at a range of depths (Jones et al., 2021). The procedure of needle positioning, voluntary contraction and signal recording was repeated until a minimum of six recordings at 25% from varying depths had been obtained to ensure sampling from a representative set of MUs. iEMG signals were amplified (D440, Digitimer, Welwyn Garden City, UK), acquired and bandpass filtered from 10 Hz to 10 kHz and sampled at 50 kHz (1401, Cambridge Electronic Design). The force and EMG signals were displayed in real‐time using Spike2 software (v9.06), and data were stored for off‐line analysis. The needle insertion site was recorded and matched from pre‐ to post‐intervention.

### 2.3 Data analysis

#### 2.3.1 HD‐sEMG analysis

HD‐sEMG data was analysed in MATLAB (v2019a, IBM) using custom written scripts to decompose and identify MUs using an extensively validated method (Martinez‐Valdes et al., 2017; Negro et al., 2016). HD‐sEMG data from the two middle contractions (contraction 3 and 4 out of six recorded) at 25% and from contraction 2 and 3 at 40% MVC were analysed. The HD‐sEMG recording electrodes remained in place during the intervention to improve the probability of sampling the same MUs. The accuracy of the decomposition for each MU was tested with the silhouette (SIL) measure, which is a normalised accuracy index for sEMG decomposition (Negro et al., 2016). Only MUPs with a SIL greater than 0.90 were included for further analysis. Subsequently, the decomposition accuracy was improved by manual editing of consecutive firings (Afsharipour et al., 2020; Boccia et al., 2019). Mean FR and the coefficient of variation for the interspike interval (FR variability) from recruited MUs were calculated from the HD‐sEMG signals from the sustained force section of the contractions. Following decomposition, individual MUs were tracked by a previously validated technique based on cross‐correlation of single‐differential 2D MUPs (Martinez‐Valdes et al., 2017). In this procedure, matched MUPs between pre‐ and post‐fatigue trials were visually inspected, and the two identified MU were regarded as the same when they had a cross‐correlation coefficient >0.80.

#### 2.3.2 iEMG analysis

**FIGURE 1:**
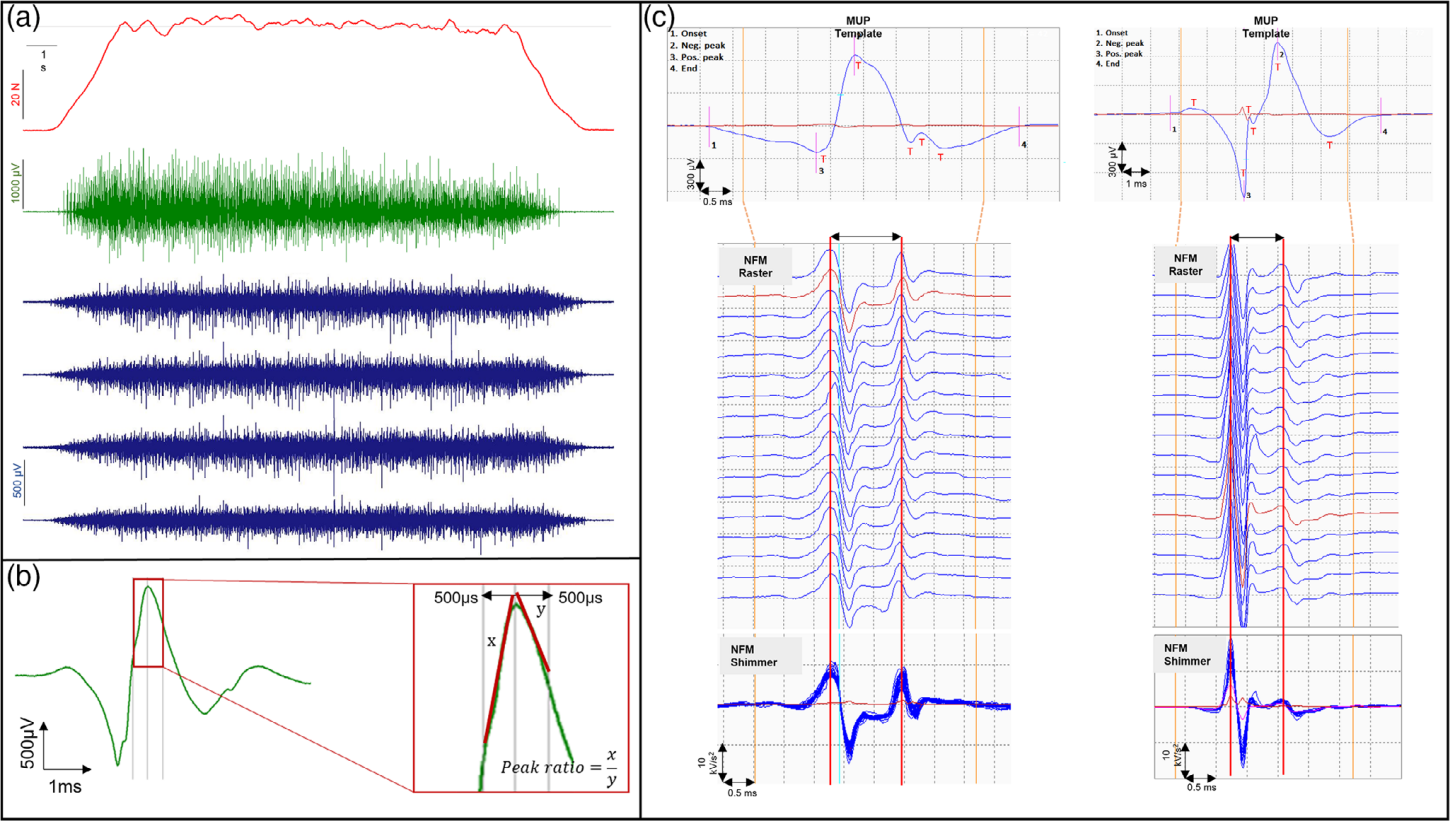
(a) Example force trace at 25% MVC (red) with corresponding iEMG (green) and subset of 4 HD‐sEMG channels (blue). (b) Representative MUP template indicating negative peak slope ratio. (c) Representative MUP templates indicating MUP turns (T) and corresponding near‐fibre MUPs (NFMs) shown in raster and shimmer plots. Abbreviations: MUP, motor unit potential; NFM, near fibre motor unit potential.

The procedures for recording and analysing individual MUPs have been described in detail previously (Piasecki, Ireland, Coulson et al., 2016; Piasecki, Ireland, Stashuk et al., 2016). Intramuscular signals from 25% contractions only were analysed using decomposition‐based quantitative electromyography (DQEMG) (Stashuk, 1999b). DQEMG was used to automatically extract MU potential trains (MUPTs) of separate MUs from an iEMG signal and calculate a MUP template for each extracted MUPT. MUPTs composed of MUPs from more than one MU or with fewer than 30 MUPs were excluded. MUP templates of included MUPTs were visually inspected and markers corresponding to the onset, end, and positive and negative peaks adjusted where required. MUP duration, in ms, is the time interval between the MUP template onset and end, MUP area, in μVms, is the MUP template area between its onset and end, MUP amplitude, in mV, is the difference between the MUP template positive and negative peak values, and MUP thickness is MUP area divided by MUP amplitude and describes the shape of the MUP template (Abdelmaseeh et al., 2014). MUP complexity was assessed using the number of turns in the MUP template. A ‘turn’ was defined as a change in direction of the MUP template of at least 25 μV and indicates the level of temporal dispersion across individual muscle fibre contributions to a single MUP. MUP negative peak slope ratio was calculated as the absolute value of the rise of the MUP template negative peak, across the 500 μs interval before the negative peak, divided by the fall of MUP template negative peak, across the 500 μs interval after the peak (Figure 1b). While the MUP template negative peak rise and fall slopes will each be similarly influenced by the relative location of the recording electrode, their ratio can represent relative rates of ion exchange during the depolarisation and repolarisation phases of an action potential, respectively. A near fibre MUP (NFM) is calculated by applying a low pass, second‐order differential filter to its corresponding MUP, which effectively reduces the recording area of the needle electrode, ensuring only the nearest fibres significantly contribute to the NFM and reducing interference from distant active fibres of other MUs (Piasecki et al., 2021; Stashuk, 1999a). All NFMs were visually inspected and those containing contamination from other NFMs were removed. NFM jiggle is a measure of the shape variability of consecutive NFMs of an MUPT expressed as a percentage of the NFM template total area (Piasecki et al., 2021) and is representative of NMJ transmission instability (Figure 1c).

#### 2.3.3 Statistical analysis

Two‐way repeated measures analysis of variance (ANOVA) with Šidák's *post hoc* analysis were performed in GraphPad Prism (v9.2, GraphPad Sofware, San Diego, CA, USA) to test for the effect of time and contraction modality on MVC and CoV force. Where no significant contraction modality × time interaction was found, main effects are reported. As multiple MUPs were recorded from each participant, multi‐level mixed effects linear regression models were performed in StataSE (v15.0, StataCorp LLC, College Station, TX, USA) with contraction modality and time as factors and contraction × time interactions included in each model. In each model the first level (individual MUs) was clustered according to each participant to form the second level. This modelling framework is suitable for data of this nature as it incorporates all sampled MUs as opposed to only the mean values obtained from each participant, which preserves variability to a greater extent within and across participants simultaneously. Regression coefficients (β) and 95% confidence intervals (CI) are reported that indicate the magnitude and direction of the effects of interest. Significance was assumed if *P* < 0.05.

## 3 RESULTS

### 3.1 Participant characteristics

Participant characteristics (6 M/6 F) are presented in Table 1.

**TABLE 1:** Participant characteristics (*n* = 12).

	Mean (SD)
Age (years)	21 (0.6)
Height (m)	1.74 (0.11)
Weight (kg)	71.9 (13.5)
BMI (kg/m^2^)	23.7 (3.4)

### 3.2 Effect of fatigue on the MU population

#### 3.2.1 Functional properties

**FIGURE 2:**
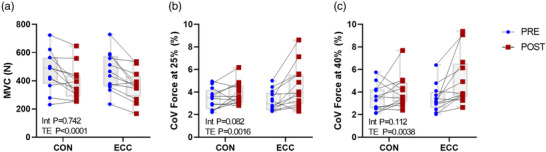
Individual maximum voluntary contraction (MVC) (a) and force steadiness at 25% (b) and 40% MVC (c) before (PRE, blue) and after (POST, red) concentric (CON) and eccentric (ECC) loading. Interaction (Int) and effect of time (TE) from two‐way ANOVAs are shown.

There was no significant contraction modality × time interaction (*P* = 0.742) for MVC but there was a main effect of time (*P* < 0.0001; Figure 2a), with MVC decreasing approximately 15.8% with CON (Pre vs. Post: 473 ± 143 N vs. 386 ± 126 N) and 20.6% with ECC (463 ± 142 N vs. 363 ± 109 N).

No significant contraction modality × time interaction for force steadiness was observed at 25% MVC but there was a main effect of time (*P* = 0.0016; Figure 2b) with a 21.7% increase with CON (3.51 ± 0.95% vs. 4.09 ± 0.95%) and a 48.8% increase with ECC (3.34 ± 0.99% vs. 5.04 ± 1.96%). No significant contraction modality × time interaction for force steadiness was observed at 40% MVC but a main effect of time was observed (*P* = 0.0038; Figure 2c) with a 20.9% increase with CON (3.71 ± 1.26% vs. 4.22 ± 1.51%) and a 56.1% increase with ECC (3.65 ± 1.34% vs. 5.45 ± 2.38%).

#### 3.2.2 MU firing

**FIGURE 3:**
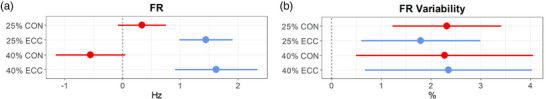
Motor unit firing rate (FR) (a) and FR variability (b) forest plots showing β and 95% confidence intervals at 25% (top) and 40% maximum voluntary contraction (MVC) (bottom) following concentric (CON) and eccentric (ECC) exercise. Statistical analyses are based on multilevel mixed effects linear models.

A total of 881 MUs were sampled with HD‐sEMG, with a mean of 7 ± 2 per 25% contraction per leg, and a mean of 6 ± 2 per 40% contraction per leg. At 25% MVC there was a significant contraction modality × time interaction for FR (*P* < 0.001), with no difference following CON (β *=* 0.34, *P* = 0.112) but a significant increase following ECC (β *=* 1.44, *P* < 0.001). Similarly, at 40% MVC there was a significant contraction modality × time interaction (*P* < 0.001) for FR, with no significant change in the CON limb (β *=* −0.55, *P* = 0.072), but an increase in the ECC limb (β *=* 1.62, *P* < 0.001) (Table 2, Figure 3a). There was no significant contraction modality × time interaction for FR variability at 25% MVC (*P* = 0.526), although it was higher post‐exercise at 25% MVC (CON; β *=* 2.32, *P* < 0.001, ECC; β *=* 1.79, *P* = 0.004). At 40% MVC there was also no significant contraction modality × time interaction (*P* = 0.902) for FR variability, with an increase in both the CON leg (β *=* 2.27, *P* = 0.002) and the ECC leg (β *=* 2.35, *P* = 0.006) (Table 2, Figure 3b).

**TABLE 2:** Group means, regression coefficient (β) and 95% confidence interval (CI) for all HD‐sEMG derived MU features (*n* = 10).

	Mean (SD)				
	PRE	POST	β	95% CI	*P*
FR (Hz) – 25%					
CON	7.73 (1.23)	8.00 (1.53)	0.34	−0.08, 0.75	0.112
ECC	7.71 (1.02)	8.78 (2.33)	**1.44**	**0.98, 1.90**	**<0.001**
FR (Hz) – 40%					
CON	8.99 (1.84)	8.82 (2.02)	−0.55	−1.15, 0.05	0.072
ECC	8.81 (1.41)	10.1 (4.08)	**1.62**	**0.91, 2.33**	**<0.001**
FR variability (%) – 25%					
CON	12.0 (2.02)	13.6 (2.20)	**2.32**	**1.22, 3.41**	**<0.001**
ECC	12.7 (2.36)	14.5 (3.20)	**1.79**	**0.589, 2.99**	**0.004**
FR variability (%) – 40%					
CON	13.8 (2.16)	16.1 (3.18)	**2.27**	**0.49, 4.05**	**0.002**
ECC	13.8 (2.49)	15.5 (5.02)	**2.35**	**0.67, 4.03**	**0.006**

Significant values shown in bold. CON, concentric; ECC, eccentric; FR, firing rate.

#### 3.2.3 Tracked MUs

**FIGURE 4:**
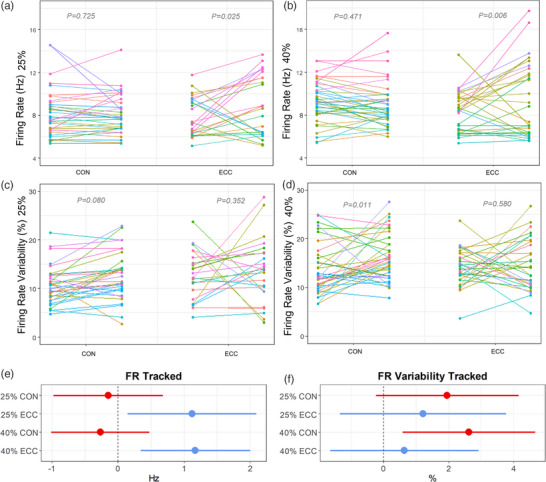
(a–d) Motor unit firing rate at 25% maximal voluntary contraction (MVC) (a) and 40% MVC (b) of individual tracked motor units for before and after concentric (CON) and eccentric (ECC) exercise, and motor unit firing rate variability at 25% MVC (c) and at 40% MVC (d). Points are colour coded for individual participants. (e, f) Motor unit firing rate (e) and firing rate variability (f) forest plots showing β and 95% confidence intervals for the change in CON and ECC exercise at both 25% (top) and 40% MVC (bottom) for the tracked MUs, from pre‐ to post‐fatiguing exercise. Statistical analyses are based on multi‐level mixed effects linear models.

From HD‐sEMG, 129 MUs (approx. 15%) were tracked from pre‐ to post‐fatiguing exercise across 10 participants. At 25% MVC there was no significant contraction modality × time interaction for FR (*P* = 0.053), and no difference in FR was observed in the CON leg following fatigue (β *=* −0.15, *P* = 0.725; Figure 4a). However, with ECC FR increased at 25% (β *=* 1.12, *P* = 0.025, Table 3, Figure 4a). There was a significant contraction modality × time interaction in FR at 40% MVC (*P* = 0.011). There was no difference in FR in the CON leg following fatigue (β *=* −0.27, *P* = 0.471; Table 3; Figure 4b); however, as at 25%, with ECC FR increased at 40% MVC (β *=* 1.17, *P* = 0.006; Figure 4b, e). Additionally, in these tracked MUs, there was no significant contraction modality × time interaction for FR variability at 25% (*P* = 0.663) with FR variability not significantly changing in CON (β *=* 1.96, *P* = 0.08) or ECC (β *=* 1.21, *P* = 0.352) following fatigue (Figure 4c). At 40% there was no significant contraction modality × time interaction (*P* = 0.203) and FR variability increased in the CON leg (β *=* 2.62, *P* = 0.011) but did not change in the ECC leg (β = 0.64, *P* = 0.580; Table 3, Figure 4d, f).

**TABLE 3:** Regression coefficient (β) and 95% confidence interval (CI) for all HD‐sEMG derived MU features in tracked units (*n* = 10).

	β	95% CI	*P*
FR (Hz) – 25%			
CON	−0.15	−0.98, 0.68	0.725
ECC	**1.12**	**0.14, 2.09**	**0.025**
FR (Hz) – 40%			
CON	−0.27	−1.01, 0.47	0.471
ECC	**1.17**	**0.34, 2.00**	**0.006**
FR variability (%) – 25%			
CON	1.96	−0.23, 4.15	0.080
ECC	1.21	−1.34, 3.76	0.352
FR variability (%) – 40%			
CON	**2.62**	**0.588, 4.65**	**0.011**
ECC	0.64	−1.63, 2.91	0.580

Significant values shown in bold. CON, concentric; ECC, eccentric; FR, firing rate.

### 3.3 Peripheral MU adaptations

**FIGURE 5:**
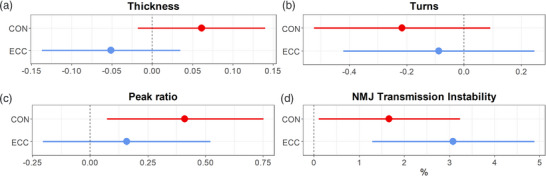
Motor unit potential (MUP) thickness (a), MUP turns (b), MUP negative peak slope ratio (c) and near fibre motor unit potential (NFM) features of neuromuscular junction (NMJ) transmission instability (d) forest plots showing β and 95% confidence intervals for the change after concentric (CON) and eccentric (ECC) exercise. Statistical analyses are based on multi‐level mixed effects linear models.

Multi‐level mixed effects regression models showed there was no significant contraction modality × time interaction for MUP thickness (*P* = 0.059) which did not differ in the CON (β *=* 0.0611, *P* = 0.130) or ECC leg (β *=* −0.0511, *P* = 0.244) (Table 4, Figure 5a). No significant contraction modality × time interaction was observed for MUP complexity (*P* = 0.58) with no significant change with either CON (β *=* −0.216, *P* = 0.168) or ECC (β *=* −0.088, *P* = 0.605; Table 4, Figure 5b). There was no significant contraction modality × time interaction for MUP negative peak slope ratio (*P* = 0.319). With CON, MUP negative peak slope ratio significantly increased (β *=* 0.411, *P* = 0.018), but there was no change with ECC (β *=* 0.159, *P* = 0.392; Table 4, Figure 5c). Although there was no significant contraction modality × time interaction for NMJ transmission instability (*P* = 0.244), it increased in both the CON (β *=* 1.66, *P* = 0.037) and ECC legs (β *=* 3.08, *P* = 0.001) (Table 4, Figure 5d).

**TABLE 4:** Group means, regression coefficient (β) and 95% confidence interval (CI) for all iEMG derived motor unit and near fibre features (*n* = 12).

	Mean (SD)				
	PRE	POST	β	95% CI	*P*
MUP thickness					
CON	1.34 (0.41)	1.34 (0.46)	0.0611	−0.018, 0.140	0.130
ECC	1.50 (0.44)	1.48 (0.57)	−0.0511	−0.137, 0.035	0.244
MUP complexity					
CON	4.1 (1.0)	4.1 (0.8)	−0.216	−0.523, 0.091	0.168
ECC	4.0 (1.1)	3.9 (1.0)	−0.088	−0.421, 0.245	0.605
MUP neg peak slope ratio					
CON	1.87 (0.28)	2.14 (0.53)	**0.411**	**0.072, 0.751**	**0.018**
ECC	2.07 (0.72)	1.97 (0.28)	0.159	−0.204, 0.521	0.392
NMJ transmission instability (%)					
CON	14.2 (3.3)	16.0 (4.7)	**1.66**	**0.10, 3.23**	**0.037**
ECC	14.8 (1.9)	17.2 (3.3)	**3.08**	**1.29, 4.88**	**0.001**

Significant values shown in bold. CON, concentric; ECC, eccentric; MUP, motor unit potential; NMJ, neuromuscular junction.

## 4 DISCUSSION

Here we present data using intramuscular needle electrodes in combination with HD‐sEMG to investigate muscle specific responses to a targeted fatiguing intervention and demonstrate that individual MU features are mediated by contraction type. Both ECC and CON resulted in similar reductions in isometric strength and force steadiness which were matched by an increase in FR variability. The neural response differed across limbs within an individual at sub‐maximal levels, with only ECC loading resulting in an increased FR during normalised force levels, and CON loading altering fibre membrane properties to a greater extent than ECC. These findings are important in understanding neuromuscular function following exercise‐induced performance fatigue and highlight the influence of contraction type on these parameters.

The functional endurance‐based intervention utilised in this study was selected to induce performance fatigue via limiting muscle activation as opposed to contractile function (Enoka & Duchateau, 2016), and the form of movement mimics that of ascending and descending stairs, a highly functional task with translational implications to ageing and disease. Both forms of contraction elicited similar decreases in maximal isometric strength, which supports similar previous findings in this muscle group (Molinari et al., 2006). Neuromuscular control, as assessed by force steadiness at normalised force levels across limbs, deteriorated in both legs. The ability to hold a steady contraction with minimal fluctuation requires effective neuromodulation of motor output (Enoka & Farina, 2021), and FR variability also increased following both ECC and CON, at both contraction intensities.

MU FR increased following ECC but did not change following CON, when assessed at 25% and 40% of MVC. Contrasting findings of FR have been observed during ECC and CON contractions in previous studies (Altenburg et al., 2008; Linnamo et al., 2003), but here we demonstrate different FR responses during an identical isometric task, after an ECC and CON fatiguing intervention. In a recent unilateral crossover study, tracked MUs increased their FR to a greater degree following CON when compared to ECC, again occurring in a contraction‐level specific manner (Hirono et al., 2022). However, these MU FRs were calculated across a broad range of contraction levels during ramped contractions, making the studies difficult to compare, and fatigue was induced by machine‐based exercise as opposed to the functional task applied here. Previous findings also show muscle twitch force reduced to a greater extent following ECC, when compared to CON contractions (Piitulainen et al., 2011), and the marked increase in FR following ECC observed in the current study likely occurs as a central mechanism to compensate for declines in twitch force (Pincheira et al., 2021). These findings are also supported by the observations of Piitulainen et al. (2012), where FR increased during sustained contractions following ECC exercise.

From the smaller sample of MUs tracked following fatigue, the differences in firing properties generally followed a similar pattern to the population results, showing an increase in FR in ECC but no significant change in CON. However, FR variability increased in CON at 40% MVC only following fatigue, but no statistical differences were observed at the lower intensity or after ECC in these tracked units. The percentage of MUs tracked is lower than previously reported and may reflect the methodological approach (e.g., HD electrodes remaining in place during intervention), an alteration of MUP duration as a result of fatigue (McManus et al., 2017) or differences in MU populations recruited immediately post‐fatigue (Martinez‐Valdes et al., 2017). Although previous research has found moderate to high level recruitment thresholds to decrease following concentric and eccentric fatiguing exercise (Hirono et al., 2022), the relatively low proportion of MUs able to be tracked prevents robust reporting of this in the current study.

The simultaneous use of iEMG enabled the collection of more detailed information on peripheral MU parameters following differing loading strategies. MUP thickness describes the shape and size of a MUP and no changes were observed following either exercise modality. The number of turns of a MUP is an assessment of MUP complexity and reflects increased temporal dispersion across MU propagating action potentials. It is applied in clinical settings to investigate myopathic and neuropathic conditions alongside other MU parameters (Allen et al., 2015; De Carvalho et al., 2014), and increases acutely following limb immobilisation (Inns et al., 2022; Sarto et al., 2022). However, similar to MUP thickness, complexity did not change following CON or ECC exercise. NF jiggle is a measure of the variability of temporal dispersion across MU propagating action potentials occurring primarily due to NMJ transmission variability (Piasecki et al., 2021), and this increased following both contraction modes. Although identifying pre‐ and post‐synaptic NMJ dysregulation in response to fatigue is extremely difficult in humans, physiological plausibility exists relative to both sites; data from animal models has shown a depletion of synaptic vesicles following prolonged stimulation mimicking fatigue (Wu & Betz, 1998), and increased exposure to acetylcholine (ACh) can result in ACh receptor desensitisation (Magleby & Pallotta, 1981). These distinct differences in NF jiggle have also been observed across age (Hourigan et al., 2015; Piasecki et al., 2020) and in disease (Allen et al., 2015), but as a result of MU remodelling functionally affecting the NMJ (Jones et al., 2022), which is unlikely to have occurred acutely in the current study. Notably, NMJ transmission instability increased at a lower absolute force (normalised to 25% post‐fatigue MVC), which is the opposite of the expected trend of increasing with larger forces (Guo et al., 2022).

The MUP negative peak slope ratio quantifies the relationship between the rise and fall slopes of the negative peak of the MUP template, reflecting the ratio of ionic exchange rates across the depolarisation and repolarisation phases of the action potential, respectively. This ratio increased following CON exercise only, which was explained by a shorter *rise* (depolarisation) time relative to the *fall* time (repolarisation), the latter of which is achieved via the efflux of intracellular K^+^. Repeated activation of skeletal muscle results in a *net* efflux of K^+^, and thus an increase in extracellular K^+^ concentration in fatigued muscle (Allen et al., 2008; Fortune & Lowery, 2009). In this fatigued state, K^+^ is required to move up its concentration gradient and may explain the slowing of repolarisation (relative to depolarisation) occurring in the limb with higher metabolic cost (the CON leg).

Collectively, these findings of adaptation of MU features following fatiguing contractions as a direct result of contraction modalities has implications for interventional strategies intended to target neural input and MU function. This knowledge may influence the contraction type employed for training depending on the requirements of the targeted population. This may also have translational relevance in understanding conditions such as sarcopenia and cancer where exercise tolerance due to muscle fatigue and weakness present a challenge in strength training (Gault & Willems, 2013).

### 4.1 Limitations

The fatiguing exercise protocol employed here required CON and ECC contractions performed to self‐reported exhaustion, but it is not possible to directly quantify the contribution of each contraction type to total exhaustion. We explored MU features up to 40% of MVC only, and further contraction‐level specific adaptations may be present at higher contractions where MUs with MU fibre type proportions may differ. Here, we were not investigating possible muscle damage effects, and therefore changes to contractile properties were not assessed. The possible influence of sex on these parameters is unclear and we did not control for hormonal status in females. However, whilst this may be viewed as a mechanistic limitation, we believe the full exclusion of females in studies of this nature would be more limiting and our previous work has found no differences in neuromuscular recruitment strategies between sexes (Guo et al., 2022).

### 4.2 Conclusion

The current findings highlight how MU features respond to fatigue in a contraction‐dependent manner, and how despite similar reductions in strength after fatiguing exercise, MU FR responded opposingly following concentric and eccentric contractions. The instability of NMJ transmission increased following both exercise modes, but markers of fibre membrane excitability were altered following the more metabolically demanding CON loading only, which likely reflects an accumulation of extracellular K^+^ and a slowing of fibre repolarisation. Differences in the MU response to concentric and eccentric induced fatigue are important when considering exercise training protocols, particularly in populations with musculoskeletal limitations.

## AUTHOR CONTRIBUTIONS

Eleanor J. Jones, Philip J. Atherton, Bethan E. Phillips and Mathew Piasecki contributed to the conception and design of the work. Eleanor J. Jones and Yuxiao Guo acquired the data. Eleanor J. Jones, Yuxiao Guo, Eduardo Martinez‐Valdes and Mathew Piasecki analysed the data. Eleanor J. Jones and Mathew Piasecki drafted the manuscript and prepared the figures. Eleanor J. Jones, Yuxiao Guo, Eduardo Martinez‐Valdes, Francesco Negro, Daniel W. Stashuk and Mathew Piasecki contributed to the interpretation of the results. All authors have read and approved the final version of this manuscript and agree to be accountable for all aspects of the work in ensuring that questions related to the accuracy or integrity of any part of the work are appropriately investigated and resolved. All persons designated as authors qualify for authorship, and all those who qualify for authorship are listed.

## CONFLICT OF INTEREST

The authors declare no conflicts of interest.

## FUNDING INFORMATION

M.P., P.J.A., B.E.P. and E.J.J. are supported by the Medical Research Council (grant number MR/P021220/1) as part of the MRC‐Versus Arthritis Centre for Musculoskeletal Ageing Research awarded to the Universities of Nottingham and Birmingham, and by the NIHR Nottingham Biomedical Research Centre.

## Supporting information

Statistical Summary Document

## Data Availability

The data that support the findings of this study are available from the corresponding author upon reasonable request.

## References

[eph13350-bib-0001] Abdelmaseeh, M. , Smith, B. , & Stashuk, D. (2014). Feature selection for motor unit potential train characterization. Muscle and Nerve, 49(5), 680–690. 23893614 10.1002/mus.23977

[eph13350-bib-0002] Adam, A. , & De Luca, C. J. (2005). Firing rates of motor units in human vastus lateralis muscle during fatiguing isometric contractions. Journal of Applied Physiology, 99(1), 268–280. 16036904 10.1152/japplphysiol.01344.2004

[eph13350-bib-0003] Afsharipour, B. , Manzur, N. , Duchcherer, J. , Fenrich, K. F. , Thompson, C. K. , Negro, F. , Quinlan, K. A. , Bennett, D. J. , & Gorassini, M. A. (2020). Estimation of self‐sustained activity produced by persistent inward currents using firing rate profiles of multiple motor units in humans. Journal of Neurophysiology, 124(1), 63–85. 32459555 10.1152/jn.00194.2020 PMC7474459

[eph13350-bib-0004] Allen, D. G. , Lamb, G. D. , & Westerblad, H. (2008). Skeletal muscle fatigue: Cellular mechanisms. Physiological Reviews, 88(1), 287–332. 18195089 10.1152/physrev.00015.2007

[eph13350-bib-0005] Allen, M. D. , Stashuk, D. W. , Kimpinski, K. , Doherty, T. J. , Hourigan, M. L. , & Rice, C. L. (2015). Increased neuromuscular transmission instability and motor unit remodelling with diabetic neuropathy as assessed using novel near fibre motor unit potential parameters. Clinical Neurophysiology, 126(4), 794–802. 25240249 10.1016/j.clinph.2014.07.018

[eph13350-bib-0006] Altenburg, T. M. , De Ruiter, C. J. , Verdijk, P. W. L. , Van Mechelen, W. , & De Haan, A. (2008). Vastus lateralis surface and single motor unit EMG following submaximal shortening and lengthening contractions. Applied Physiology, Nutrition and Metabolism, 33(6), 1086–1095. 10.1139/H08-092 19088766

[eph13350-bib-0007] Boccia, G. , Martinez‐Valdes, E. , Negro, F. , Rainoldi, A. , & Falla, D. (2019). Motor unit discharge rate and the estimated synaptic input to the vasti muscles is higher in open compared with closed kinetic chain exercise. Journal of Applied Physiology, 127(4), 950–958. 31369324 10.1152/japplphysiol.00310.2019

[eph13350-bib-0008] Burtin, C. , Saey, D. , Saglam, M. , Langer, D. , Gosselink, R. , Janssens, W. , Decramer, M. , Maltais, F. , & Troosters, T. (2012). Effectiveness of exercise training in patients with COPD: The role of muscle fatigue. European Respiratory Journal, 40(2), 338–344. 22135284 10.1183/09031936.00111811

[eph13350-bib-0009] Chaudhuri, A. , & Behan, P. O. (2004). Fatigue in neurological disorders. Lancet, 363(9413), 978–988. 15043967 10.1016/S0140-6736(04)15794-2

[eph13350-bib-0010] Christie, A. , Snook, E. M. , & Kent‐Braun, J. A. (2011). Systematic review and meta‐analysis of skeletal muscle fatigue in old age. Medicine and Science in Sports and Exercise, 43(4), 568–577. 20881888 10.1249/MSS.0b013e3181f9b1c4 PMC3705929

[eph13350-bib-0011] Contessa, P. , De Luca, C. J. , & Kline, J. C. (2016). The compensatory interaction between motor unit firing behavior and muscle force during fatigue. Journal of Neurophysiology, 116(4), 1579–1585. 27385798 10.1152/jn.00347.2016 PMC5144693

[eph13350-bib-0012] Cook, C. J. , Beaven, C. M. , & Kilduff, L. P. (2013). Three weeks of eccentric training combined with overspeed exercises enhances power and running speed performance gains in trained athletes. Journal of Strength and Conditioning Research, 27(5), 1280–1286. 22820207 10.1519/JSC.0b013e3182679278

[eph13350-bib-0013] Dartnall, T. J. , Rogasch, N. C. , Nordstrom, M. A. , & Semmler, J. G. (2009). Eccentric muscle damage has variable effects on motor unit recruitment thresholds and discharge patterns in elbow flexor muscles. Journal of Neurophysiology, 102(1), 413–423. 19420118 10.1152/jn.91285.2008

[eph13350-bib-0014] De Carvalho, M. , Turkman, A. , & Swash, M. (2014). Sensitivity of MUP parameters in detecting change in early ALS. Clinical Neurophysiology, 125(1), 166–169. 23845892 10.1016/j.clinph.2013.06.014

[eph13350-bib-0015] Del Vecchio, A. , Negro, F. , Felici, F. , & Farina, D. (2018). Distribution of muscle fibre conduction velocity for representative samples of motor units in the full recruitment range of the tibialis anterior muscle. Acta Physiologica, 222(2), e12930. 10.1111/apha.12930 28763156

[eph13350-bib-0016] Duchateau, J. , & Baudry, S. (2014). Insights into the neural control of eccentric contractions. Journal of Applied Physiology, 116(11), 1418–1425. 23429873 10.1152/japplphysiol.00002.2013

[eph13350-bib-0017] Enoka, R. M. , & Duchateau, J. (2008). Muscle fatigue: What, why and how it influences muscle function. The Journal of Physiology, 586(1), 11–23. 17702815 10.1113/jphysiol.2007.139477 PMC2375565

[eph13350-bib-0018] Enoka, R. M. , & Duchateau, J. (2016). Translating fatigue to human performance. Medicine and Science in Sports and Exercise, 48(11), 2228–2238. 27015386 10.1249/MSS.0000000000000929 PMC5035715

[eph13350-bib-0019] Enoka, R. M. , & Duchateau, J. (2017). Rate coding and the control of muscle force. Cold Spring Harbor Perspectives in Medicine, 7(10), a029702. 28348173 10.1101/cshperspect.a029702 PMC5629984

[eph13350-bib-0020] Enoka, R. M. , & Farina, D. (2021). Force steadiness: From motor units to voluntary actions. Physiology, 36(2), 114–130. 33595382 10.1152/physiol.00027.2020

[eph13350-bib-0021] Fortune, E. , & Lowery, M. M. (2009). Effect of extracellular potassium accumulation on muscle fiber conduction velocity: A simulation study. Annals of Biomedical Engineering, 37(10), 2105–2117. 19588250 10.1007/s10439-009-9756-4

[eph13350-bib-0022] Gault, M. L. , & Willems, M. E. T. (2013). Aging, functional capacity and eccentric exercise training. Aging and Disease, 4(6), 351–363. 24307968 10.14336/AD.2013.0400351 PMC3843652

[eph13350-bib-0023] Guo, Y. , Jones, E. J. , Inns, T. B. , Ely, I. A. , Stashuk, D. W. , Wilkinson, D. J. , Smith, K. , Piasecki, J. , Phillips, B. E. , Atherton, P. J. , & Piasecki, M. (2022). Neuromuscular recruitment strategies of the vastus lateralis according to sex. Acta Physiologica, 235(2), e13803. 35184382 10.1111/apha.13803 PMC9286427

[eph13350-bib-0024] Hirono, T. , Kunugi, S. , Yoshimura, A. , Holobar, A. , & Watanabe, K. (2022). Acute changes in motor unit discharge property after concentric versus eccentric contraction exercise in knee extensor. Journal of Electromyography and Kinesiology, 67, 102704. 36137408 10.1016/j.jelekin.2022.102704

[eph13350-bib-0025] Hody, S. , Croisier, J. L. , Bury, T. , Rogister, B. , & Leprince, P. (2019). Eccentric muscle contractions: Risks and benefits. Frontiers in Physiology, 1, 1–18. 10.3389/fphys.2019.00536 PMC6510035 31130877

[eph13350-bib-0026] Hourigan, M. L. , McKinnon, N. B. , Johnson, M. , Rice, C. L. , Stashuk, D. W. , & Doherty, T. J. (2015). Increased motor unit potential shape variability across consecutive motor unit discharges in the tibialis anterior and vastus medialis muscles of healthy older subjects. Clinical Neurophysiology, 126(12), 2381–2389. 25727901 10.1016/j.clinph.2015.02.002

[eph13350-bib-0027] Hunter, S. K. , Duchateau, J. , & Enoka, R. M. (2004). Muscle fatigue and the mechanisms of task failure. Exercise and Sport Sciences Reviews, 32(2), 44–49. 15064647 10.1097/00003677-200404000-00002

[eph13350-bib-0028] Inns, T. B. , Bass, J. J. , Hardy, E. J. O. , Wilkinson, D. J. , Stashuk, D. W. , Atherton, P. J. , Phillips, B. E. , & Piasecki, M. (2022). Motor unit dysregulation following 15 days of unilateral lower limb immobilisation. The Journal of Physiology, 600(21), 4753–4769. 36088611 10.1113/JP283425 PMC9827843

[eph13350-bib-0029] Jones, E. J. , Chiou, S.‐Y. , Atherton, P. J. , Phillips, B. E. , & Piasecki, M. (2022). Ageing and exercise‐induced motor unit remodelling. The Journal of Physiology, 600(8), 1839–1849. 35278221 10.1113/JP281726 PMC9314090

[eph13350-bib-0030] Jones, E. J. , Piasecki, J. , Ireland, A. , Stashuk, D. W. , Atherton, P. J. , Phillips, B. E. , McPhee, J. S. , & Piasecki, M. (2021). Lifelong exercise is associated with more homogeneous motor unit potential features across deep and superficial areas of vastus lateralis. GeroScience, 43(4), 1555–1565. 33763775 10.1007/s11357-021-00356-8 PMC8492837

[eph13350-bib-0031] Kay, D. , St Clair Gibson, A. , Mitchell, M. J. , Lambert, M. I. , & Noakes, T. D. (2000). Different neuromuscular recruitment patterns during eccentric, concentric and isometric contractions. In Journal of Electromyography and Kinesiology. pp. 425–431. Elsevier. 10.1016/s1050-6411(00)00031-6 11102845

[eph13350-bib-0032] Kostek, M. C. , Chen, Y. W. , Cuthbertson, D. J. , Shi, R. , Fedele, M. J. , Esser, K. A. , & Rennie, M. J. (2007). Gene expression responses over 24 h to lengthening and shortening contractions in human muscle: Major changes in CSRP3, MUSTN1, SIX1, and FBXO32. Physiological Genomics, 31(1), 42–52. 17519359 10.1152/physiolgenomics.00151.2006

[eph13350-bib-0033] Kuchinad, R. , Ivanova, T. , & Garland, S. J. (2004). Modulation of motor unit discharge rate and H‐reflex amplitude during submaximal fatigue of the human soleus muscle. Experimental Brain Research, 158(3), 345–355. 15146306 10.1007/s00221-004-1907-0

[eph13350-bib-0034] Linnamo, V. , Bottas, R. , & Komi, P. V. (2000). Force and EMG power spectrum during and after eccentric and concentric fatigue. Journal of Electromyography and Kinesiology, 10(5), 293–300. 11018439 10.1016/s1050-6411(00)00021-3

[eph13350-bib-0035] Linnamo, V. , Moritani, T. , Nicol, C. , & Komi, P. V. (2003). Motor unit activation patterns during isometric, concentric and eccentric actions at different force levels. Journal of Electromyography and Kinesiology, 13(1), 93–101. 12488091 10.1016/s1050-6411(02)00063-9

[eph13350-bib-0036] Magleby, K. L. , & Pallotta, B. S. (1981). A study of desensitization of acetylcholine receptors using nerve‐released transmitter in the frog. The Journal of Physiology, 316(1), 225–250. 6275065 10.1113/jphysiol.1981.sp013784 PMC1248799

[eph13350-bib-0037] Martinez‐Valdes, E. , Laine, C. M. , Falla, D. , Mayer, F. , & Farina, D. (2016). High‐density surface electromyography provides reliable estimates of motor unit behavior. Clinical Neurophysiology, 127(6), 2534–2541. 26778718 10.1016/j.clinph.2015.10.065

[eph13350-bib-0038] Martinez‐Valdes, E. , Negro, F. , Laine, C. M. , Falla, D. , Mayer, F. , & Farina, D. (2017). Tracking motor units longitudinally across experimental sessions with high‐density surface electromyography. The Journal of Physiology, 595(5), 1479–1496. 28032343 10.1113/JP273662 PMC5330923

[eph13350-bib-0039] McManus, L. , Hu, X. , Rymer, W. Z. , Suresh, N. L. , & Lowery, M. M. (2017). Motor unit activity during fatiguing isometric muscle contraction in hemispheric stroke survivors. Frontiers in Human Neuroscience, 11, 569. 29225574 10.3389/fnhum.2017.00569 PMC5705653

[eph13350-bib-0040] Merletti, R. , Farina, D. , Gazzoni, M. , & Schieroni, M. P. (2002). Effect of age on muscle functions investigated with surface electromyography. Muscle & Nerve, 25(1), 65–76. 11754187 10.1002/mus.10014

[eph13350-bib-0041] Molinari, F. , Knaflitz, M. , Bonato, P. , & Actis, M. V. (2006). Electrical manifestations of muscle fatigue during concentric and eccentric isokinetic knee flexion‐extension movements. IEEE Transactions on Bio‐Medical Engineering, 53(7), 1309–1316. 16830935 10.1109/TBME.2006.873680

[eph13350-bib-0042] Negro, F. , Muceli, S. , Castronovo, A. M. , Holobar, A. , & Farina, D. (2016). Multi‐channel intramuscular and surface EMG decomposition by convolutive blind source separation. Journal of Neural Engineering, 13(2), 026027. 26924829 10.1088/1741-2560/13/2/026027

[eph13350-bib-0043] Oliveira, A. S. , & Negro, F. (2021). Neural control of matched motor units during muscle shortening and lengthening at increasing velocities. Journal of Applied Physiology, 130(6), 1798–1813. 33955258 10.1152/japplphysiol.00043.2021

[eph13350-bib-0044] Overend, T. J. , Versteegh, T. H. , Thompson, E. , Birmingham, T. B. , & Vandervoort, A. A. (2000). Cardiovascular stress associated with concentric and eccentric isokinetic exercise in young and older adults. Journals Gerontol ‐ Series A, Biological Sciences and Medical Sciences, 55(4), B177–B182. 10.1093/gerona/55.4.b177 10811144

[eph13350-bib-0045] Peñailillo, L. , Blazevich, A. , Numazawa, H. , & Nosaka, K. (2013). Metabolic and muscle damage profiles of concentric versus repeated eccentric cycling. Medicine and Science in Sports and Exercise, 45(9), 1773–1781. 23475167 10.1249/MSS.0b013e31828f8a73

[eph13350-bib-0046] Piasecki, J. , Inns, T. B. , Bass, J. J. , Scott, R. , Stashuk, D. W. , Phillips, B. E. , Atherton, P. J. , & Piasecki, M. (2020). Influence of sex on the age‐related adaptations of neuromuscular function and motor unit properties in elite masters athletes. The Journal of Physiology, 599(1), 193–205. 33006148 10.1113/JP280679

[eph13350-bib-0047] Piasecki, M. , Garnés‐Camarena, O. , & Stashuk, D. W. (2021). Near‐fiber electromyography. Clinical Neurophysiology, 132(5), 1089–1104. 33774377 10.1016/j.clinph.2021.02.008

[eph13350-bib-0048] Piasecki, M. , Ireland, A. , Coulson, J. , Stashuk, D. W. , Hamilton‐Wright, A. , Swiecicka, A. , Rutter, M. K. , McPhee, J. S. , & Jones, D. A. (2016). Motor unit number estimates and neuromuscular transmission in the tibialis anterior of master athletes: Evidence that athletic older people are not spared from age‐related motor unit remodeling. Physiological Reports, 4(19), e12987. 27694526 10.14814/phy2.12987 PMC5064139

[eph13350-bib-0049] Piasecki, M. , Ireland, A. , Piasecki, J. , Degens, H. , Stashuk, D. W. , Swiecicka, A. , Rutter, M. K. , Jones, D. A. , & McPhee, J. S. (2019). Long‐term endurance and power training may facilitate motor unit size expansion to compensate for declining motor unit numbers in older age. Frontiers in Physiology, 10, 449. 31080415 10.3389/fphys.2019.00449 PMC6497749

[eph13350-bib-0050] Piasecki, M. , Ireland, A. , Stashuk, D. , Hamilton‐Wright, A. , Jones, D. A. , & McPhee, J. S. (2016). Age‐related neuromuscular changes affecting human vastus lateralis. The Journal of Physiology, 594(16), 4525–4536. 26486316 10.1113/JP271087 PMC4983624

[eph13350-bib-0051] Piitulainen, H. , Botter, A. , Merletti, R. , & Avela, J. (2011). Muscle fiber conduction velocity is more affected after eccentric than concentric exercise. European Journal of Applied Physiology, 111(2), 261–273. 20865423 10.1007/s00421-010-1652-y

[eph13350-bib-0052] Piitulainen, H. , Holobar, A. , & Avela, J. (2012). Changes in motor unit characteristics after eccentric elbow flexor exercise. Scandinavian Journal of Medicine & Science in Sports, 22(3), 418–429. 20973828 10.1111/j.1600-0838.2010.01228.x

[eph13350-bib-0053] Pincheira, P. A. , Martinez‐Valdes, E. , Guzman‐Venegas, R. , Falla, D. , Garrido, M. I. , Cresswell, A. G. , & Lichtwark, G. A. (2021). Regional changes in muscle activity do not underlie the repeated bout effect in the human gastrocnemius muscle. Scandinavian Journal of Medicine & Science in Sports, 31(4), 799–812. 33378553 10.1111/sms.13912

[eph13350-bib-0054] Prinsen, H. , Van Dijk, J. P. , Zwarts, M. J. , Leer, J. W. H. , Bleijenberg, G. , & Van Laarhoven, H. W. M. (2015). The role of central and peripheral muscle fatigue in postcancer fatigue: A randomized controlled trial. Journal of Pain and Symptom Management, 49(2), 173–182. 25150812 10.1016/j.jpainsymman.2014.06.020

[eph13350-bib-0055] Roig, M. , O'Brien, K. , Kirk, G. , Murray, R. , McKinnon, P. , Shadgan, B. , & Reid, W. D. (2009). The effects of eccentric versus concentric resistance training on muscle strength and mass in healthy adults: A systematic review with meta‐analysis. British Journal of Sports Medicine, 43(8), 556–568. 18981046 10.1136/bjsm.2008.051417

[eph13350-bib-0056] Rubinstein, S. , & Kamen, G. (2005). Decreases in motor unit firing rate during sustained maximal‐effort contractions in young and older adults. Journal of Electromyography and Kinesiology, 15(6), 536–543. 16054395 10.1016/j.jelekin.2005.04.001

[eph13350-bib-0057] Sarto, F. , Stashuk, D. W. , Franchi, M. V. , Monti, E. , Zampieri, S. , Valli, G. , Sirago, G. , Candia, J. , Hartnell, L. M. , Paganini, M. , McPhee, J. S. , De Vito, G. , Ferrucci, L. , Reggiani, C. , & Narici, M. V. (2022). Effects of short‐term unloading and active recovery on human motor unit properties, neuromuscular junction transmission and transcriptomic profile. The Journal of Physiology, 600(21), 4731–4751. 36071599 10.1113/JP283381 PMC9828768

[eph13350-bib-0058] Souron, R. , Nosaka, K. , & Jubeau, M. (2018). Changes in central and peripheral neuromuscular fatigue indices after concentric versus eccentric contractions of the knee extensors. European Journal of Applied Physiology, 118(4), 805–816. 29411127 10.1007/s00421-018-3816-0

[eph13350-bib-0059] Stashuk, D. W. (1999a). Detecting single fiber contributions to motor unit action potentials. Muscle and Nerve, 22(2), 218–229. 10024135 10.1002/(sici)1097-4598(199902)22:2<218::aid-mus10>3.0.co;2-s

[eph13350-bib-0060] Stashuk, D. W. (1999b). Decomposition and quantitative analysis of clinical electromyographic signals. Medical Engineering & Physics, 21(6–7), 389–404. 10624736 10.1016/s1350-4533(99)00064-8

[eph13350-bib-0061] Stock, M. S. , Beck, T. W. , & Defreitas, J. M. (2012). Effects of fatigue on motor unit firing rate versus recruitment threshold relationships. Muscle & Nerve, 45(1), 100–109. 22190315 10.1002/mus.22266

[eph13350-bib-0062] Webber, S. , & Kriellaars, D. (1997). Neuromuscular factors contributing to in vivo eccentric moment generation. Journal of Applied Physiology, 83(1), 40–45. 9216942 10.1152/jappl.1997.83.1.40

[eph13350-bib-0063] Wu, L.‐G. , & Betz, W. J. (1998). Kinetics of synaptic depression and vesicle recycling after tetanic stimulation of frog motor nerve terminals. Biophysical Journal, 74(6), 3003–3009. 9635754 10.1016/S0006-3495(98)78007-5 PMC1299641

